# Morphological Characterization and Genotyping of *Acanthamoeba* Isolates From Oral and Nasal Samples of Cancer Patients in Kashan, Iran

**DOI:** 10.1155/2024/4071707

**Published:** 2024-11-13

**Authors:** Sima Rasti, Tayebeh Taghipour, Mahdi Delavari, Hossein Hooshyar, Gholam Abbas Moosavi, Mohsen Arbabi

**Affiliations:** ^1^Infectious Diseases Research Center, Kashan University of Medical Sciences, Kashan, Isfahan, Iran; ^2^Department of Parasitology & Mycology, School of Medicine, Kashan University of Medical Sciences, Kashan, Isfahan, Iran; ^3^Department of Statistics and Public Health, School of Health, Kashan University of Medical Sciences, Kashan, Isfahan, Iran

**Keywords:** *Acanthamoeba*, cancer patients, genotyping, nasal, oral cavity

## Abstract

**Background:** *Acanthamoeba* species are recognized as the most prevalent free-living amoebae (FLA). They can cause granulomatous amebic encephalitis (GAE) and pulmonary and ocular infections. The present study aimed to isolate and identify *Acanthamoeba* genotypes in cancer patients referred to Kashan's hospitals in Central Iran.

**Methods:** This cross-sectional study was conducted with oral and nasal swab samples collected from a hundred cancer patients referred to Kashan's Beheshti and Yasrebi hospitals during 2019–2020. The samples were cultured in 1.5% non-nutrient agar (NNA) with heat-killed *Escherichia coli* and examined for “FLA.” A polymerase chain reaction (PCR) assay amplifying the 18S rRNA gene was performed, and *Acanthamoeba*-positive isolates were subjected to nucleotide sequencing to identify their genotypes.

**Results:** The prevalence of *Acanthamoeba* infection was 51% in the oral cavity and 38% in the nasal samples of cancer patients. The most frequent *Acanthamoeba* cysts were (51.3%) wrinkled polygonal and sized 9.55-11.5 μm (Group II). *Acanthamoeba* genotypes T4, T11, and T5 were identified in the oral cavity samples, whereas T4 and T11 were detected in the nasal samples.

**Conclusion:** The prevalence of *Acanthamoeba* infection in oral and nasal cancer patients was higher in Kashan, Iran, compared to other countries. Due to the high rate of oral *Acanthamoeba* contamination, oral sampling is recommended for better detection of this protozoan. Since T4 is the predominant genotype that can cause serious diseases in high-risk groups, increasing physicians' awareness of infections associated with *Acanthamoeba* and preventive and control measures are strongly suggested.

## 1. Introduction

*Acanthamoeba* is one of the most common free-living amoebae (FLA) in the environment. This opportunistic protozoan can be found in various environments including soil, hospital dust, fresh and saltwater, hot water, swimming pools, air conditioning systems, and seawater, posing a risk of infection during water activities [1–6]. It has also been isolated from dental units, dialysis machines, and hospital environments [3, 7, 8]. In a study by Bradbury, French, and Blizzard, *Acanthamoeba* was isolated from two endotracheal tube samples of a patient [9]. The potential pathogenic role of *Acanthamoeba* has been emphasized in patients receiving respiratory support in intensive care units (ICUs) [9]. Bacteria, such as *Legionella pneumophila*, *Mycobacterium avium*, *Helicobacter pylori*, *Escherichia coli*, and *Klebsiella pneumoniae,* can live symbiotically in the cytoplasm of *Acanthamoeba* and increase the pathogenicity of bacteria and amoebae [10–12]. Intracellular bacterial microbiomes of *Acanthamoeba* strains, such as *Pseudomonas* species and *Enterobacteriales*, were reported in Australian and Indian corneal isolates [13]. This parasite hosts a wide range of bacteria and trains them to become more pathogenic [14].

Kot, Łanocha-Arendarczyk, and Kosik-Bogacka reported 19 cases of *Acanthamoeba* pneumonia in immunocompromised patients (i.e., leukemia patients and recipients of liver, kidney, lung, and heart transplantation) in the United States, Poland, Australia, India, Japan, South Korea, and France [15]. There are a few opportunistic protozoa, such as *Acanthamoeba*, which are responsible for granulomatous amebic encephalitis (GAE), pulmonary infections, ocular keratitis, skin ulcers, and disseminated infections in individuals with immunodeficiency or immunosuppressed patients including cancer patients undergoing chemotherapy and transplant recipients [3, 16–18]. Many of these infections can be fatal or can lead to life-threatening complications if not diagnosed and treated promptly [3]. Even in developed countries with various diagnostic facilities, diagnosis is often only done postmortem through autopsy [19].

*Acanthamoeba* keratitis (AK) is an increasingly common infectious cornea disease. The incidence of AK ranges from 17 to 70 cases per million contact lens wearers annually. The risk factors for this disease include wearing contact lenses, exposure to contaminated water or hands, and ocular trauma [20].

*Acanthamoeba* genotypes are identified through sequencing assays [7, 21]. *Acanthamoeba* species can be identified based on the diagnostic fragment 3 (DF3) region of 18S rRNA. So far, 23 *Acanthamoeba* genotypes (T1–T23) have been detected [22, 23]. In a previous study, the prevalence of pathogenic *Acanthamoeba* genotypes was estimated at 26% in nasal swab samples and 12.2% in the salivary samples of cancer patients in Tehran, Iran; T4 was found to be the predominant genotype [24, 25]. T4 is also more prevalent in keratitis-infected patients; however, a few T3, T5, T9, T11, and T15 cases have been reported [26].

Due to the high level of dust contamination (52.5%) with the pathogenic T4 genotype of *Acanthamoeba* in Kashan hospitals [2] and the lack of information about *Acanthamoeba* infection in patients undergoing chemotherapy, the present study aims to determine *Acanthamoeba* genotypes in cancer patients, referred to two hospitals (Shahid Beheshti and Yasrebi) in Kashan, Iran.

## 2. Materials and Methods

### 2.1. Ethics Statement

This study was approved by the Ethics Committee of the Kashan University of Medical Sciences, Kashan, Iran (Code: IR.KAUMS.MEDNT.REC.1398.056).

### 2.2. Sampling Area

Kashan is a city in the northwest of Isfahan Province, Iran. It has a hot desert climate with cold winters and stormy winds. The average temperature is 19°C, with a maximum of 50°C.

### 2.3. Population Study

This cross-sectional study was conducted on 100 cancer patients undergoing chemotherapy for 3–6 months in Shahid Beheshti and Yasrebi subspecialty hospitals of Kashan, Iran, from 2019 to 2020.

### 2.4. Oral and Nasal Cavity Sampling

After obtaining informed consent from the patients to participate in the study, their demographic information was recorded in a questionnaire. Two sterile swab samples (QC Isan Teb, Iran) were simultaneously collected from the oral and nasal cavities. Samples were collected from the end of the oral cavity, between the teeth, and the opening of the pharynx, using a swab moistened with a sterile physiological saline. Samples were placed in sterile tubes and transferred to the parasitology laboratory.

### 2.5. Isolation of FLA

#### 2.5.1. Sample Preparation

First, 2 mL of sterile physiological saline was poured into tubes containing patients' mucosal samples and kept at laboratory temperature (28°C–30°C) for three days. Next, the sample tube was vortexed until the mucus adhered to the swab, and the physiological saline was thoroughly mixed, forming a suspension [27].

#### 2.5.2. Mass Cultivation

Oral mucosal suspensions and nasal swabs were separately cultured on 1.5% NNA culture medium (BACTO Agar, DIFCO, United States of America), enriched with heat-killed *Escherichia coli*, and placed in an incubator (Teif Azma Teb, Iran) at 30°C–32°C [7, 8, 27, 28]. After 3 days, the plates were examined daily or weekly for 2 months under an inverted microscope (Tension, China) to investigate the presence of trophozoites or cysts of “FLA.” If a trophozoite or *Acanthamoeba* cyst was observed, the sample was considered positive and passaged. Meanwhile, if no parasites were detected, the sample would be recorded negative. In the present study, the modified culturing procedure was conducted as follows.

**Figure 1:**
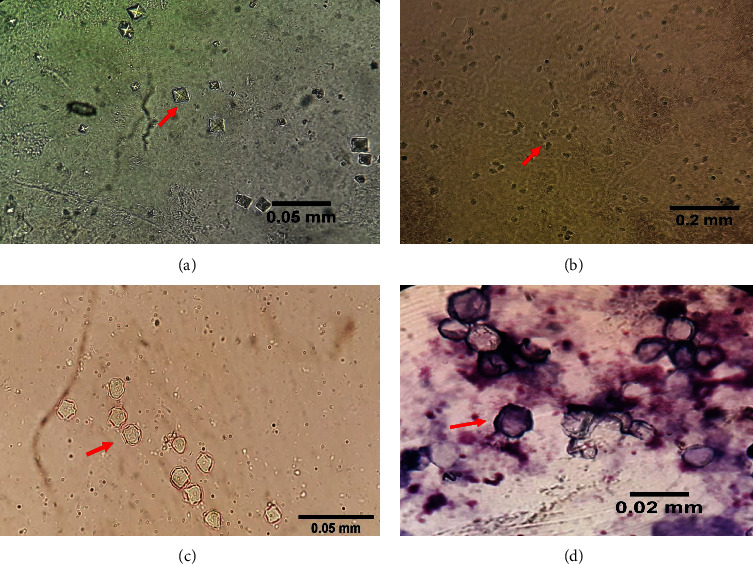
Morphology of trophozoites and *Acanthamoeba* cysts (arrows) isolated from the oral cavity samples of cancer patients in Kashan, Iran. (a) Star-shaped *Acanthamoeba* cysts in the culture medium after 45 days (400× magnification). (b) *Acanthamoeba* trophozoite (100× magnification). (c) Polygonal-shaped *Acanthamoeba* cysts (400× magnification). (d) *Acanthamoeba* cyst (Giemsa staining, 1000× magnification).

3 mL of sterile physiological saline and 200 *μ*L of heat-killed *E. coli* suspension were added to the dried plates to ensure a moistened culture surface. Then, by observation of the star-shaped cyst of *Acanthamoeba* (Figure 1(a)) and moisturizing the culture, it converted to trophozoite and cyst [27, 29, 30]. In order to reduce bacterial and fungal contamination and improve the identification of “FLA,” wherever “FLA” was seen on the plate they were marked, a single colony was transferred using a swab moistened with sterile physiological saline and placed into enriched NNA containing heat-killed *E. coli*. The sample was sealed and then incubated at 30°C–32°C. Following the passage of positive samples, a pure mass culture was prepared. After pouring 3 mL of physiological saline on the surface plate, cysts can be scraped off the agar, transferred into a sterile tube, and centrifuged at 2500 rpm for 2 min. In the final step, the “FLA” sediment was kept at −20°C until DNA extraction [2, 27, 30].

#### 2.5.3. Morphological Identification

To determine the morphological characteristics of “FLA” cysts, wet smears were prepared and examined under a light microscope. The smears were also studied via Giemsa staining and observed under a microscope at 100X, 400X, and 1000X. Finally, the morphology of *Acanthamoeba* was determined according to Pussard's criteria. Based on the shape and size of *Acanthamoeba* cyst, there are three morphological groups including Group I: size more than 18 *μ*m and polyclonal shape, II: less than 18 *μ*m and polyclonal, and III: less than 18 *μ*m and round shape [31].

### 2.6. PCR and Genotyping

The DNA of positive samples was extracted using a DNP kit (CinnaGen Co., Iran), according to the manufacturer's instructions. Subsequently, PCR assay (Flex Cycler2, Germany) was conducted with *Acanthamoeba*-specific primers: JDP1 (5′GGCCCAGATCGTTTACCGTGAA-3′) and JDP2 primers (5′TCTCACAAGCTGCTAGGGAGTCA-3′) on the DF3 region of 18S rRNA gene at an annealing temperature of 61.2°C [24, 27]. A positive control (*Acanthamoeba*) and a negative control (sterile distilled water) were used in each PCR reaction. After electrophoresis of the PCR product, *Acanthamoeba* was identified by observing the approximately 500 bp band adjacent to the 100 bp marker. In this study, a total of 16 oral cavity isolates and 15 nasal isolates were sequenced. Using the Basic Local Alignment Search Tool (BLAST on the NCBI website) for nucleotide sequences, the genotypes of *Acanthamoeba* were identified and recorded in the DNA Data Bank of Japan (DDBJ).

### 2.7. Phylogenetic Analyses

This study used the Molecular Evolutionary Genetic Analysis Version 7 (MEGA7) software for phylogenetic analyses of DF3 sequences. *Acanthamoeba culbertsoni*, *Acanthamoeba castellanii*, and *Acanthamoeba polyphaga* were selected as reference strains and *Hartmannella* sp. and *Entamoeba histolytica* as out group strains.

### 2.8. Data Analysis

After recording the collected data in SPSS version 16.0 (SPSS Inc., Chicago, Illinois, United States of America), statistical analysis was performed by measuring descriptive statistics and conducting Fisher's exact tests. The relationship between these statistics and the patient's demographic characteristics was also analyzed.

## 3. Results

### 3.1. Demographical Data and Cancer Types of Patients

In this study, the mean age of the patients was 54.2 ± 14 years (minimum, 19 years; maximum, 82 years). In terms of cytology, out of 100 cancer patients undergoing chemotherapy, gastrointestinal cancer (i.e., esophageal, stomach, small intestine, large intestine, rectal, and gall bladder carcinomas; *n* = 33, 33%) was the most prevalent carcinoma. This was followed by breast cancer (29%) and leukemia (10%), whereas the rates of testicular cancer and abdominal tumor were the lowest (*n* = 3 per group).

### 3.2. Frequency of FLA and *Acanthamoeba* by Culture and PCR

**Figure 2:**
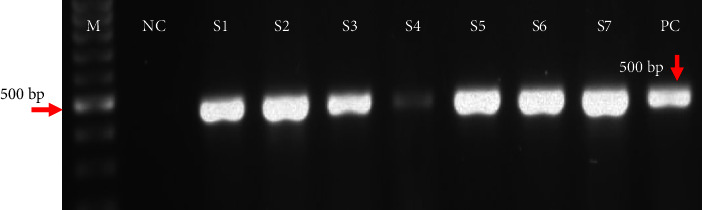
Gel electrophoresis of approximately 500 bp PCR product of *Acanthamoeba* sp. using JDP1 and JDP2 primers, isolated from oral cavity samples of cancer patients in Kashan hospitals, Iran (NC: negative control; S1–S7: positive *Acanthamoeba* samples; M: marker 100 bp; PC: positive control).

Out of 100 oral cavity samples, 89 tested positive for “FLA,” and 11 negative in the culture. Among the 89 positive samples*, Acanthamoeba* was confirmed in 51 samples using the PCR assay (Figure 2), while 9 were negative for *Acanthamoeba*. Due to the low “FLA” count in 29 cultured samples, DNA extraction and PCR were not performed on them. Additionally, *Acanthamoeba* was positive in 38 out of 100 nasal samples.

The prevalence of *Acanthamoeba* infection was 69.6% in females and 44.2% in males, and the difference was statistically significant (*p*=0.05). The rate of infection was 71.9% in the age group of < 50 years (*p*=0.265), which was not statistically significant (Table 1). Based on the findings, out of 51 cancer patients who tested positive for *Acanthamoeba*, five (55.6%) had a history of smoking, and nine (56.3%) had diabetes.

### 3.3. Distribution of *Acanthamoeba* According to the Types of Cancer

**Figure 3:**
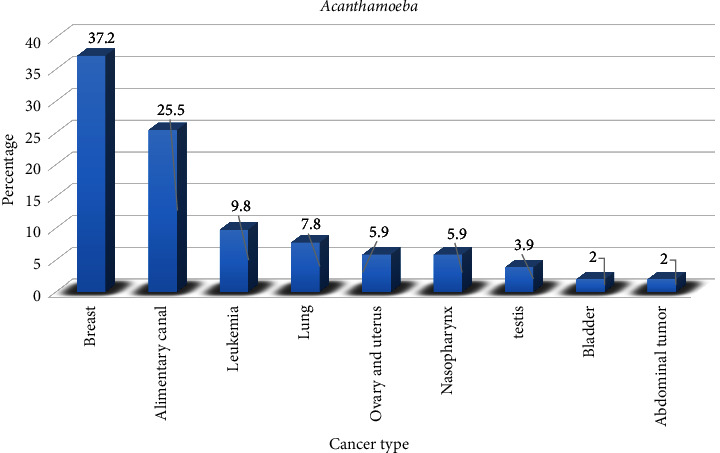
Distribution of 51 oral cavity samples from cancer patients infected with *Acanthamoeba* according to the type of cancer.

Out of 51 oral cavity samples collected from cancer patients who were infected with *Acanthamoeba*, the highest prevalence of contamination was reported in breast (37.2%) and alimentary canal cancer (25.5%). At the same time, the lowest rate was found in patients with bladder cancer and abdominal masses (2%) (Figure 3).

### 3.4. Genotyping and Morphological Analyses of *Acanthamoeba*

The genotypes of 31 *Acanthamoeba* isolates from cancer patients' oral cavity and nasal samples were determined. Among 31 *Acanthamoeba* isolates, 28 (90.3%) belonged to T4, two (6.5%) to T11, and one (3.2%) belonged to the T5 genotype (Table 2).

After sequencing and genotyping *Acanthamoeba*, the samples were submitted to DDBJ (Gene Bank), and their accession number and genetic similarity were identified. Furthermore, the shape and size of *Acanthamoeba* were determined.

The *Acanthamoeba* morphological characteristics indicates that out of 76 isolated cysts, 51.3% were wrinkled polygonal cysts and 48.71% were round smooth, respectively (*p*=0.909). However, among 39 wrinkled polygonal cysts, mostly 20 (51.3%) tend to have size 9.55–11.5 *μ*m (*p*=0.014) (Table 3).

Genotype, accession number, genetic similarity, and morphological groups of *Acanthamoeba* isolated from oral (Table 4) and nasal samples (Table 5) in the cancer patients are mentioned, respectively.

Figure 1. shows Acanthamoeba trophozoites and cysts isolated from oral cavity samples of cancer patients. (a) Star-shaped cyst, (b) trophozoite, and (c and d) polygonal cyst.

### 3.5. Phylogenetic Analyses

**Figure 4:**
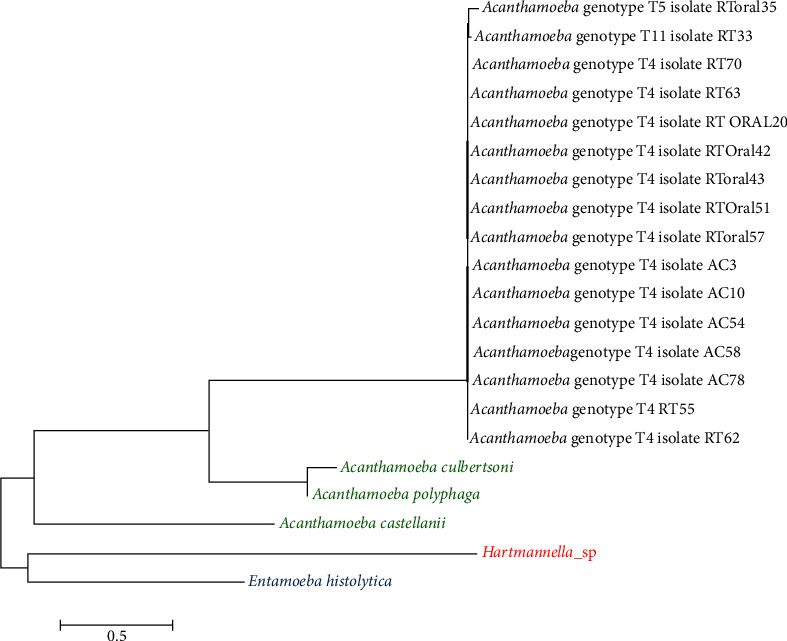
Phylogenetic tree of oral isolated *Acanthamoeba* sp. based on 18S rRNA with the nearest validated species with high bootstrap value. *Acanthamoeba* isolates were shown in black color, reference strains *Acanthamoeba culbertsoni*, *A. castellanii*, and *A. polyphaga* in green color, and out group strains *Hartmannella* sp. and *Entamoeba histolytica* in red and blue colors, respectively.

**Figure 5:**
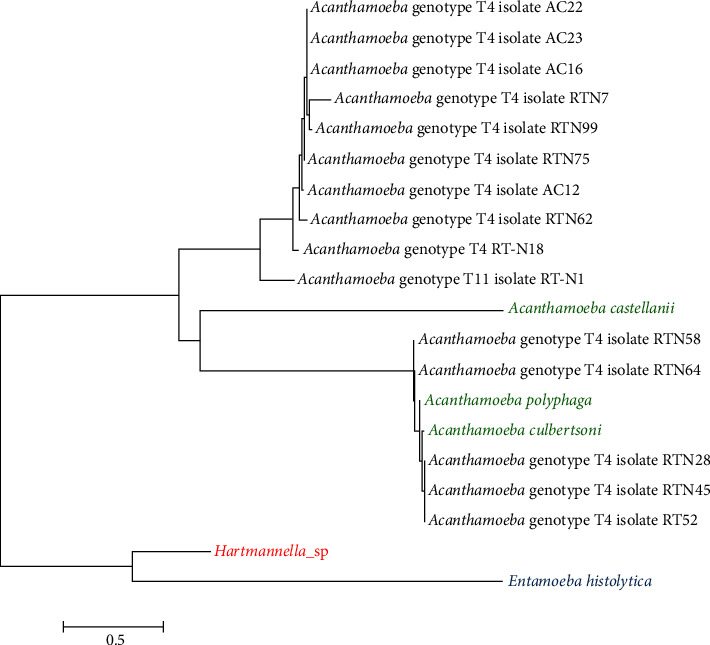
Phylogenetic tree of nasal isolated *Acanthamoeba* sp. based on 18S rRNA with the nearest validated species with high bootstrap value. *Acanthamoeba* isolates were shown in black color, reference strains *Acanthamoeba culbertsoni*, *A. castellanii*, and *A. polyphaga* in green color, and out group strains *Hartmannella* sp. and *Entamoeba histolytica* in red and blue colors, respectively.

Phylogenetic relationships between *Acanthamoeba* are inferred from DNA of partial 18S rRNA collected from oral and nasal cancer patients in Kashan, Iran, which showed that all the samples have similar genetic characterization (Figures 4 and 5).

The phylogenetic tree of *Acanthamoeba* isolated from the oral (Figure 4) and nasal cavity (Figure 5) in cancer patients is shown, respectively.

## 4. Discussion

Findings of the present study revealed that the rate of contamination with “FLA” and *Acanthamoeba* was 89% and 51% in the oral cavity and 89% and 38% in the nasal swabs of cancer patients in Kashan, Iran, respectively, which exceeded the rates reported by previous researchers. The rate of *Acanthamoeba* was reported as 6.3% in Philippian street sweepers and 1.4% in the low-exposure group [32], 28.4% in healthy people in Peru [33], 13.4% in oral samples of immunocompromised patients in Tehran [25], 26.3% in nasal samples of cancer patients [8], and 10% in oral cavity of immunocompromised patients in Tabriz, Iran [34], respectively. The contamination rate of these protozoa in AIDS and post-heart transplant patients was reported as 7.1% and 7.5%, respectively [35, 36]. Using the morphological method, Pezeshki, Haniloo, and Mahmoodzadeh reported an infection rate of 6% with *Acanthamoeba* in the nasal swab samples of cancer patients in Zanjan, Iran [37]. Overall, microscopic and morphological methods are not accurate enough for detecting *Acanthamoeba*.

The higher prevalence of *Acanthamoeba* in the present study can be attributed to using modified techniques for cultivating and harvesting “FLA.” This process involves incubating oral and nasal swabs in physiological saline for 2 days before cultivation. The samples were then separated from the swabs through vortexing, and freshly heat-killed *E. coli* was used as the food source for *Acanthamoeba*. The samples were cultivated at an appropriate temperature for 2 months, followed by frequent passages and plate examinations [27]. Overall, the combination of these processes can effectively reach more accurate results about the rate of “FLA” and identifying *Acanthamoeba* species; it also justifies the high level of contamination found in this study.

Based on the results of a study by Taghipour et al. on the bronchoalveolar lavage (BAL) samples of suspected cancer patients in Kashan, the rate of contamination with “FLA” and *Acanthamoeba* was 89% and 73.2%, respectively, which is consistent with the results of the present study [27].

In another study by Golestani et al., the prevalence of *Acanthamoeba* contamination in dust, soil, and stagnant water was 52.5%, 62.5%, and 50% in Beheshti Hospital of Kashan, Iran, respectively. Additionally, the prevalence of water contamination with *Acanthamoeba* was 11.7% in rural areas of Kashan [2, 38]. The rate of soil contamination was 100% in the Philippines [32].

Moreover, in the present study, the high contamination rate with “FLA” and *Acanthamoeba* in the oral cavity samples of cancer patients is probably related to the climatic conditions of Kashan (desert region and seasonal dust storms). The results of studies by Golestani et al. and Mustafaei et al. confirm this finding [2, 38].

According to the previous study, *Acanthamoeba* cysts are highly resistant and can be alive and transmitted to people, especially immunocompromised patients (cancer patients) by dust and air pollution and airborne [2, 8, 30]. That may indicate the high prevalence of *Acanthamoeba* in the present study. Furthermore, the high prevalence (28.4%) of this parasite in healthy people [33] shows that this parasite is commensal. So, the higher rate of *Acanthamoeba* in cancer patients (high-risk group) is legitimate.

Interestingly, the increasing rate of *Acanthamoeba* in the oral cavity (51%) compared to the nasal cavity (39%) may be due to salivary secretions, moisture in the oral cavity, and normal microbial flora. The aforementioned factors provide suitable conditions for the growth and nutrition of *Acanthamoeba* [39].

In the present study, *Acanthamoeba* genotypes belonged to T4, T5, and T11 in the oral cavity and T4 and T11 in the nasal samples of cancer patients (Tables 2, 4, and 5). Previous studies reported T4, T3, and T5 genotypes in immunocompromised patients' nasal and oral samples [8, 24, 32, 35]. Niyyati and Rezaeian reported T4, T2, T3, T5, T6, and T11 in environmental and clinical samples [40]. In addition, Golestani et al. identified T4, T5, T2, T7, and T11 genotypes from environmental sources in Kashan, Iran. The genotypes of the present study revealed similarity to *Acanthamoeba* isolates collected from dust in the Beheshti hospital environment [2]. Hajialilo et al. detected T4, T9, and T11 in keratitis patients [41], consistent with the present results.

In the present study, T4 was the predominant *Acanthamoeba* genotype in all clinical samples. T11 and T5 genotypes were identified in the oral and nasal cavity samples, respectively (Tables 2, 4, and 5) consistent with previous studies' results [24, 40, 41].

Moreover, in this study, the highest rates of *Acanthamoeba* infection were reported in women (69.6%), individuals under 50 years of age (71.9%), those with no education or only elementary school education (59.3%), and housewives (69.8%) (Table 1). In a study by Mathin, Ismail, and Mehmood, a high level of anti-*Acanthamoeba* antibodies was found in people aged 25–30 years, which is consistent with the results of the present study [42]. Additionally, the frequency of wrinkled polygonal *Acanthamoeba* cysts (different sizes) was statistically significant compared to round smooth form in the samples of cancer patients (*p*=0.014) (Table 3).

## 5. Conclusion

The present study indicated a relatively high prevalence of *Acanthamoeba* in cancer patients, especially in oral cavity samples. In this study, the dominant genotype of the isolates was the T4 genotype, which is highly pathogenic and responsible for severe sequelae, such as GAE and keratitis patients. Therefore, it should not be neglected in patients with malignancies, and screening for diagnosis of this disease is recommended. Additionally, assessment of pathogenicity factors and drug studies on *Acanthamoeba* isolates are suggested.

## Figures and Tables

**Table 1:** Distribution of *Acanthamoeba* and “FLA” in oral cavity samples of cancer patients according to demographic characteristics, Kashan, Iran.

Risk factors	Variables	*N* = 89	*Acanthamoeba*-positive cases *N* (%)	*Acanthamoeba*-negative cases *N* (%)	Free-living amoeba *N* (%)	*p* value
Sex	Female	46	32 (69.6)	3 (6.5)	11 (23.9)	0.05∗
	Male	43	19 (44.2)	6 (14)	18 (41.9)	

Age	< 50	32	23 (71.9)	3 (9.4)	6 (18.8)	0.265∗
	51–65	39	19 (48.7)	4 (10.3)	16 (41)	
	> 65	18	9 (50)	2 (11.1)	7 (38.9)	

Education level	Illiterate	59	35 (59.3)	8 (13.6)	16 (27.1)	0.301∗
	Diploma	20	11 (55)	0 (0)	9 (45)	
	Academic	10	5 (50)	1 (10)	4 (40)	

Job	Housekeeper	43	30 (69.8)	2 (4.7)	11 (25.5)	0.08∗
	Employee	13	6 (46.1)	1 (7.7)	6 (46.2)	
	Farmer	9	5 (55.6)	1 (11.1)	3 (33.3)	
	Retired	11	4 (36.4)	1(9.1)	6(54.5)	
	Educational	4	3 (75)	0	1 (25)	
	Other	9	3 (33.3)	4 (44.5)	2 (22.2)	

^∗^Fisher's exact tests.

**Table 2:** Distribution of *Acanthamoeba* genotypes in cancer patients' oral cavity and nasal samples according to the type of samples, Kashan, Iran.

Type of sample	Genotype			
	T4	T11	T5	Total
Oral cavity	14 (87.5)	1 (6.25)	1 (6.25)	16 (100)
Nasal	14 (93.3)	1 (6.7)	0	15 (100)
Total	28 (90.3)	2 (6.5)	1 (3.2)	31 (100)

**Table 3:** Distribution of *Acanthamoeba* morphology according to the shape and size in cancer patients' oral cavity and nasal samples, Kashan, Iran.

Size (*μ*m)	Shape			
	Wrinkled, polygonal *N* (%)	Round, smooth N (%)	Total *N* (%)
8–9.5	6 (15.4)	17 (45.9)	23 (30.3)	*p*=0.014
9.55–11.5	20 (51.3)	13 (35.1)	33 (43.4)	
11.55–17	13 (33.3)	7 (18.9)	20 (26.3)	
Total	39 (100)	37 (100)	76 (100)	

**Table 4:** Genotype, accession number, genetic similarity, morphology, size, and morphological groups of *Acanthamoeba* isolated from the oral cavity in the cancer patients recorded in the DNA data bank.

No.	Isolate No.	Genotype	Genetic similarity (%)	Accession No.	Shape and size of cyst (*μ*m)	Morphological groups
1	AC3	T4	99.8	LC598793.1	Wrinkled, polygonal, 11.3	II
2	RT-oral20	T4	98.74	LC598505.1	Wrinkled, polygonal, 12.2	II
3	RT-oral42	T4	99.7	LC598499.1	Round, smooth, 11.9	III
4	RT-oral51	T4	100	LC598501.1	Wrinkled, polygonal, 12	II
5	AC10	T4	99.3	LC598957.1	Wrinkled, polygonal, 10.4	II
6	RT55	T4	99.76	LC598790.1	Wrinkled, polygonal, 10.4	II
7	RT33	T11	98.6	LC598500.1	Round, smooth, 9.4	III
8	RT-oral35	T5	100	LC598502.1	Wrinkled, polygonal, 14.5	II
9	RT-oral43	T4	99.53	LC598503.1	—	—
10	RT-oral57	T4	99.2	LC598504.1	Wrinkled, polygonal, 13.9	II
11	RT62	T4	99.7	LC598506.1	Wrinkled, polygonal, 10.2	II
12	RT63	T4	99.7	LC598507.1	Wrinkled, polygonal, 10.8	II
13	RT70	T4	99.3	LC598508.1	Wrinkled, polygonal, 12.4	II
14	AC54	T4	99.7	LC598795.1	Round, smooth, 9.5	III
15	AC78	T4	98.3	LC598796.1	Wrinkled, polygonal, 11.9	II
16	AC58	T4	99.7	LC598797.1	Round, smooth, 11.4	III

**Table 5:** Genotype, accession number, genetic similarity, morphology, size, and morphological groups of *Acanthamoeba* isolated from nasal samples in the cancer patients registered in the DNA databank.

No.	Isolate No.	Genotype	Genetic similarity (%)	Accession No.	Shape and size of cyst (*μ*m)	Morphological groups
1	RT-N1	T11	99.48	LC604814.1	Wrinkled, polygonal, 11.5	II
2	RT-N18	T4	98.51	LC625878.1	Round, smooth, 15	III
3	AC12	T4	99.52	LC598794.1	Round, smooth, 9.4	III
4	RT-N7	T4	89.3	LC598791.1	Wrinkled, polygonal, 9.8	II
5	AC23	T4	100	LC598956.1	—	—
6	RT-N62	T4	97.1	LC625879.1	Wrinkled, polygonal, 8.5	II
7	AC16	T4	99.76	LC598954.1	—	—
8	RT-N75	T4	96.5	LC604815.1	Round, smooth, 10.1	III
9	AC22	T4	99.77	LC598955.1	Wrinkled, polygonal, 11.1	II
10	RT-N99	T4	100	LC625877.1	Wrinkled, polygonal, 8.5	II
11	RT-N64	T4	99.5	LC626599	—	—
12	RT-52	T4	92.1	LC626600	Wrinkled, polygonal, 10	II
13	RT-N45	T4	99.5	LC626601	Round, smooth, 9.1	III
14	RT-N28	T4	99.8	LC626602	Wrinkled, polygonal, 8.8	II
15	RT-N58	T4	98.4	LC626603	Wrinkled, polygonal, 7.9	II

## Data Availability

The data that support the findings of this study are available from the corresponding author upon reasonable request.
